# Optimizing Digital Impressions: A Review of Factors Affecting Intraoral Scanner Accuracy

**DOI:** 10.3390/diagnostics16162625

**Published:** 2026-08-19

**Authors:** Gabriela Czerepak, Aleksandra Jędras, Katarzyna Bednarz, Weronika Pociask, Katarzyna Gliwa, Weronika Czajkowska, Wiktoria Zowczak, Leszek Szalewski

**Affiliations:** 1 Student Research Group at the Digital Dentistry Laboratory, Medical University of Lublin, 20-059 Lublin, Poland; gabriela.czerepak1@gmail.com (G.C.) jedrasaleksandra@gmail.com (A.J.); kat.bed00@gmail.com (K.B.); weronika.pociask10@gmail.com (W.P.); katmgliwa@gmail.com (K.G.); weroczajk@gmail.com (W.C.); w.zowczak2@gmail.com (W.Z.); 2 Digital Dentistry Laboratory, Medical University of Lublin, 20-059 Lublin, Poland; leszek.szalewski@umlub.edu.pl

**Keywords:** intraoral scanner, digital impression, scanning strategy, operator experience

## Abstract

**Background**: Digital intraoral scanners (IOSs) represent one of the key tools in contemporary digital dentistry and are widely applied in prosthodontics, orthodontics, implantology, and treatment monitoring. The aim of this study was to critically analyze the factors affecting the accuracy of intraoral scanning and to evaluate their clinical significance. **Methods**: This study was designed as a narrative literature review conducted using a structured literature search. The analysis was based on publications retrieved from the PubMed and ScienceDirect databases and published between 2016 and 2026 using keywords related to intraoral scanners, digital impressions, trueness, precision, scanning strategy, operator experience, scanner type, restorative materials, and clinical conditions. **Results**: Twenty publications selected according to the review scope and eligibility criteria were included. Based on the analysis, four major groups of factors influencing IOS accuracy were identified: scanning strategy, operator experience, scanner type and restorative material, and clinical/environmental conditions. The available evidence most consistently associated scan quality with the scanner type and the applied scanning strategy, although the effectiveness of individual protocols depended on the specific IOS system used. The influence of operator experience remained inconclusive and appeared to be less significant in modern devices equipped with advanced image reconstruction algorithms. Clinical factors such as lighting conditions, arch morphology, subgingival preparation margins, maneuverability restrictions, and material-related optical properties also played an important role, whereas humidity and moisture control should be interpreted as possible clinical confounders rather than experimentally verified independent factors in the included primary studies. **Conclusions**: The findings indicate that intraoral scanning accuracy is a multifactorial phenomenon requiring individualized adaptation of the scanning procedure to clinical conditions and the type of IOS system used. Because the included evidence was heterogeneous and did not allow for formal ranking of effect magnitude, conclusions regarding the relative importance of individual factors should be interpreted with caution.

## 1. Introduction

Digital intraoral scanners (IOSs) are among the most rapidly evolving technologies in contemporary dentistry. Introduced into clinical practice with the CEREC system in 1985, these devices have undergone multiple technological advancements—from systems requiring contrast powder application to modern high-resolution scanners equipped with advanced software algorithms [1,2]. Currently, more than 15 IOS systems are available on the market, and their progressively decreasing costs contribute to their increasing adoption in routine clinical practice [1].

Intraoral scanners replace conventional impressions made with alginate or silicone materials, enabling rapid and comfortable acquisition of a three-dimensional digital model of the dental arches. The main advantages of digital techniques include elimination of deformation errors associated with impression materials, reduced working time, and simplified data storage and transfer [1,3]. The applications of IOSs now extend far beyond prosthodontics and include orthodontics, implantology, periodontology, and treatment monitoring [2,3,4].

Despite these advantages, the accuracy of intraoral scanning remains a critical issue from a clinical perspective. Scan precision determines the quality of prosthetic restorations, occlusal splints, orthodontic appliances, and aligners fabricated on the basis of digital impressions, while deviations from the actual tissue geometry may lead to restoration misfit and the need for adjustment or remanufacturing. In the clinical context, two fundamental parameters are used to evaluate scan quality: trueness, defined as the agreement between the acquired scan and the actual geometry of the scanned object, and precision, which refers to the repeatability of results obtained from repeated scans of the same area [5]. Both parameters are clinically important, as high precision combined with low trueness does not guarantee the accuracy of the final restoration [5,6].

The accuracy of intraoral scanning is influenced by numerous interrelated factors that are generally classified into three major categories in the literature: operator-related, patient-related, and device-related factors [6]. Operator-dependent variables include experience, training level, scanning technique, scanning strategy, and familiarity with a given IOS system [7,8]. Patient-related factors involve intraoral conditions such as humidity, saliva and blood contamination, tissue morphology, depth of the preparation margin, and the presence of highly reflective restorative materials such as gold or zirconia, which may generate artifacts and image distortions [6,8]. Device-related factors include image acquisition technology, size and shape of the scanning tip, software version, and environmental conditions such as ambient lighting [6,8]. The extent of the scanned area also plays an important role [8,9].

Despite the growing number of studies focusing on IOS systems, the interactions among these factors remain insufficiently understood. Limited understanding of these relationships may directly contribute to clinical errors, restoration misfits, repeated impressions, and increased treatment costs. Furthermore, the lack of standardized protocols for evaluating scanning accuracy complicates inter-study comparisons and hinders the development of evidence-based clinical recommendations [8].

Although numerous studies have investigated individual factors affecting intraoral scanner accuracy, the available evidence remains fragmented. Most publications focus on isolated variables, such as scanner type, scanning strategy, or operator experience, while relatively few studies discuss the interactions among these determinants and their combined impact on clinical outcomes. Moreover, differences in study design, experimental protocols, and accuracy assessment methods make direct comparison difficult. Therefore, a comprehensive narrative synthesis of the available evidence is needed to provide clinicians with a practical overview of the current knowledge, identify existing research gaps, and highlight areas requiring further investigation.

The aim of this study was to critically analyze the factors affecting intraoral scanning accuracy and their interrelationships, with particular emphasis on clinical implications.

## 2. Materials and Methods

This study was conducted as a narrative review of the scientific literature concerning factors affecting the accuracy of intraoral scanners (IOSs). Relevant publications were identified through searches of the PubMed and ScienceDirect databases. The complete PubMed search string was as follows: ((“intraoral scanner”[Title/Abstract] OR “intraoral scanners”[Title/Abstract] OR “digital impression”[Title/Abstract] OR “digital impressions”[Title/Abstract]) AND (accuracy[Title/Abstract] OR trueness[Title/Abstract] OR precision[Title/Abstract] OR “scanning strategy”[Title/Abstract] OR “operator experience”[Title/Abstract] OR “scanner type”[Title/Abstract] OR “restorative material”[Title/Abstract] OR “clinical conditions”[Title/Abstract] OR lighting[Title/Abstract])).

The complete ScienceDirect search string was as follows: (“intraoral scanner” OR “intraoral scanners” OR “digital impression” OR “digital impressions”) AND (accuracy OR trueness OR precision OR “scanning strategy” OR “operator experience” OR “scanner type” OR “restorative material” OR “clinical conditions” OR lighting).

Searches were limited to English-language publications published between 1 January 2016 and 10 June 2026, which was the date of the final database search. In addition, reference lists of selected articles were manually screened to identify further relevant publications.

Following the removal of duplicate records, titles and abstracts were screened independently by three reviewers. Full-text assessment of potentially relevant publications was subsequently performed by the same reviewers. Any disagreements regarding study eligibility were resolved through discussion until consensus was reached; if necessary, the supervisor was consulted.

Original research articles and review papers addressing factors influencing intraoral scanner accuracy were eligible for inclusion. Publications were selected based on their relevance to the review question, methodological quality, and contribution to the current understanding of clinical and technical determinants of intraoral scanning accuracy. As this study was designed as a narrative review, no formal risk-of-bias assessment or quantitative meta-analysis was performed.

The available evidence was synthesized narratively, with findings organized into four major groups of factors influencing intraoral scanner performance: scanning strategy, operator experience, scanner-related factors, and clinical or environmental conditions.

## 3. Results

The literature review included 20 publications: 16 in vitro studies, three clinical investigations, and one review article. Four major groups of factors influencing intraoral scanning accuracy were identified: scanning strategy (5 studies), operator experience (4 studies), scanner type and restorative material (3 studies), and clinical/environmental conditions (8 studies). Although the individual groups were analyzed with varying levels of detail, the available evidence consistently indicated that IOS accuracy is a multifactorial phenomenon and that isolated evaluation of single variables may lead to incomplete or misleading conclusions [8] (Table 1).

### 3.1. Scanning Strategy

Among all analyzed factors, scanning strategy was one of the most extensively investigated. Ortensi et al. compared three full-arch scanning protocols (S1—manufacturer-recommended, S2—modified, and S3—experimental) using four different IOS systems and demonstrated that the selected strategy significantly affected trueness, precision, and scanning time. Strategy S2 achieved the highest precision for most devices and the shortest scanning time, whereas strategy S3 provided the highest trueness for CS 3600 and Medit i700 scanners, offering the most favorable balance among all evaluated parameters [10].

Grande et al. examined scanning accuracy in a different clinical context, namely maxillary complete-arch implant scans. In this study, implant scan body type and IOS type influenced accuracy, whereas scan strategy did not significantly affect trueness but did influence scanning efficiency [11]. This finding suggests that conclusions regarding scanning strategy should be interpreted within the specific indication and IOS system tested rather than generalized across dentate and implant-supported full-arch scenarios.

Comparable findings were reported by Răuță et al., who compared Medit i700 and Trios 5 scanners and demonstrated that both scanner type and scanning strategy significantly influenced scan accuracy. Trios 5 showed greater variability depending on the applied strategy, indicating higher sensitivity of this system to operator technique [12].

Liu et al. highlighted the relationship between scanning strategy and arch location, demonstrating that while maxillary scans yielded comparable results regardless of protocol, the OH and TQ strategies produced significantly lower deviations when scanning the mandible [13]. Similarly, Chang et al., analyzing three scanning strategies in a maxillary Kennedy Class I partially edentulous model, found no significant differences in overall accuracy among groups, although the TR strategy exhibited the most favorable distribution of measurement points [14].

### 3.2. Operator Experience

The influence of operator experience on scanning outcomes remains controversial, and available evidence is inconsistent. Sawangsri et al. confirmed a significant effect of experience on both scan accuracy and scanning time, regardless of scan pattern or arch scanned [15]. In contrast, Thomas and Jain, in a prospective clinical study involving 20 patients, demonstrated that greater experience reduced scanning time but did not significantly improve accuracy [16].

Similar conclusions were drawn by Lin et al., who evaluated 30 operators grouped according to experience level using three IOS systems: Omnicam, Primescan, and Aoralscan 3. No significant differences in accuracy were found between operator groups, whereas scanner type itself had a pronounced effect; Omnicam demonstrated lower accuracy and longer scanning time regardless of operator experience [17].

Unexpected findings were reported by Schimmel et al., whose study showed that operator experience had only a small overall influence on accuracy in completely and partially edentulous models. The apparent advantage of the inexperienced operator was limited to selected edentulous models, whereas experienced operators completed scans more quickly [18]. Overall, the available evidence suggests that operator experience consistently affects scanning efficiency, while its direct effect on accuracy is more variable and probably depends on scanner generation, clinical difficulty, and the model being scanned.

### 3.3. Scanner Type and Restorative Material

The type of intraoral scanner used is among the most consistently reported determinants of digital impression accuracy, although the available evidence does not allow for a formal hierarchy of factor importance. In the study by Lin et al., CEREC Omnicam demonstrated significantly lower accuracy and longer scanning times compared with Primescan and Aoralscan 3, regardless of operator experience [17]. Răuță et al. confirmed that Medit i700 produced lower deviations and higher precision than Trios 5 under the tested conditions [12], while Ortensi et al. demonstrated that the effectiveness of a given scanning strategy was strongly dependent on the scanner used, indicating an interaction between scanning protocol and scanner-specific reconstruction algorithms [10].

Differences among scanners become particularly evident in clinically demanding situations. Çakmak et al. demonstrated that the interaction between scanner type (Primescan and TRIOS 3) and finish-line location (subgingival, equigingival, and supragingival) significantly affected the accuracy of laminate veneer preparations, with subgingival margins yielding the lowest accuracy regardless of the scanner used [19].

The method of tooth preparation finishing also affects scanning accuracy. Revilla-León et al. demonstrated that airborne-particle abrasion provided the highest trueness and precision among all tested finishing methods, whereas immediate dentin sealing (IDS) was associated with the lowest accuracy. Among the scanners evaluated, the i700 system achieved the best trueness and precision values, whereas CS 3800 and Trios 4 produced the least favorable results [20].

The type of restorative material also significantly influences scanning accuracy. Budati et al. showed that highly reflective or translucent materials may generate greater scanning errors, and different scanning technologies vary in their ability to manage such materials, emphasizing the absence of a universally optimal solution for all clinical situations [21].

### 3.4. Clinical and Environmental Factors

Clinical, environmental, and methodological factors constitute another important group affecting IOS accuracy. Yu et al. demonstrated that scanning performance was significantly reduced under intraoral conditions compared with extraoral conditions, mainly due to prolonged scanning time and limited scanner maneuverability [22].

Similar conclusions were drawn by Kirschneck et al., who reported differences in conformity, reliability, and validity between digital dental models created by clinical intraoral scanning and extraoral plaster model digitization workflows. The study indicates that intraoral scanning may show lower reliability in selected regions, particularly where access and stitching are more challenging [23].

Arch morphology also affects scanning accuracy. Lin et al. demonstrated that severe dental crowding significantly reduced scanning trueness, likely due to impaired optical access and an increased number of undercut areas. Conversely, interdental spacing facilitated more predictable and accurate scans, suggesting that anatomical factors may substantially affect final scan quality independently of operator or device characteristics [24].

Shen et al. complemented these findings by comparing intraoral scanning with indirect methods involving silicone impressions and gypsum casts. Although the extraoral scanner demonstrated slightly lower deviations in the incisor region, no significant differences were found between methods in posterior or non-prepared areas, supporting the clinical reliability of intraoral scanning under the tested conditions [25].

The color of scanned tissues and ambient lighting conditions also influence scanning outcomes. Zhou et al. demonstrated that darker shades of zirconia restorations required additional ambient illumination to maintain comparable scanning accuracy [26]. These findings were further supported by Guo et al., who evaluated the effect of light intensity and color temperature on scan accuracy and shade matching. The authors demonstrated that illuminance affected geometric scanning accuracy, whereas color temperature mainly influenced shade matching rather than scan geometry itself. Reported optimal lighting parameters differed between TRIOS 4 and Primescan scanners [27].

Fratila et al. identified an even broader range of environmental and technical variables affecting IOS performance, including software version, scanner resolution, tissue morphology, arch width, lighting, moisture-related factors, and ambient temperature [8]. However, factors such as humidity should be interpreted cautiously in the present review because they were mainly discussed as clinical or environmental considerations and were not consistently tested as independent experimental variables in the included primary studies. An additional methodological issue concerns the method used for scan superimposition. Vargas-Quiroga et al. demonstrated that superimposition techniques based primarily on hard tissues while minimizing soft tissue inclusion provided the highest repeatability, which is particularly relevant for long-term monitoring of oral changes [28].

## 4. Discussion

The present literature review confirms that intraoral scanning accuracy is a complex phenomenon influenced by technological, human, and environmental factors. Importantly, 16 of the 19 included primary studies were in vitro, whereas only three were clinical investigations. Consequently, conclusions regarding causal effects, relative scanner performance, and procedural optimization are supported predominantly by laboratory evidence and should not be interpreted as direct proof of clinical superiority. The clinical investigations support the practical relevance of selected observations, but their limited number restricts generalizability. Because the included studies also differed in reference standards, scanner systems, clinical models, and outcome measures, the results should be interpreted as a descriptive synthesis rather than as a quantitative ranking of factor importance.

### 4.1. Influence of Scanning Strategy

Scanning strategy represents one of the most extensively investigated aspects and has been shown in several in vitro and clinical contexts to affect trueness, precision, and scanning time [10,11,12,13,14]. These findings are consistent with earlier reports by Abduo et al. and Alkadi, who emphasized the importance of scanning sequence and scanning tip trajectory for minimizing cumulative reconstruction errors [5,6]. Different strategies influence not only the distribution of local inaccuracies but also scanning time and operator workload. Importantly, the optimal scanning sequence varies among devices and indications, suggesting the need for scanner-specific protocols. Clinically, this supports adherence to manufacturer recommendations and discourages unvalidated modifications of scanning trajectories, particularly in full-arch and implant-related workflows.

### 4.2. Importance of Operator Experience

The influence of operator experience remains inconclusive. Some studies indicate a significant relationship between experience and scan trueness and precision [15], whereas others do not confirm such an association [16,17]. These discrepancies are likely related to advancements in data processing algorithms capable of compensating for technical inaccuracies introduced by less experienced users [18]. This may suggest that in modern highly automated systems, the importance of the human factor is reduced, although operator skill remains relevant in clinically demanding situations such as subgingival margin scanning, edentulous arches, implant cases, and limited-access areas. Therefore, adequate training and familiarity with the specific IOS system should remain fundamental components of digital workflow implementation.

### 4.3. Scanner Type and Reproduction Accuracy

The analyzed studies consistently identified scanner type as an important determinant of digital impression accuracy [10,12,17,26]. Differences among systems may reflect scanning technology, reconstruction algorithms, resolution, tip design, software version, and sensitivity to reflective-surface artifacts. New-generation scanners such as Primescan and Medit i700 generally showed favorable accuracy and repeatability in the analyzed comparisons; however, performance also varied according to scanning strategy, restorative material, finish-line location, and lighting conditions. The available evidence therefore does not support a universal ranking of scanners across all clinical situations [10,12,17,19,20,21,26,27].

It should also be noted that both wired scanners (e.g., wired 3Shape TRIOS 3 and Carestream CS 3600 connected via USB) and wireless systems (e.g., 3Shape TRIOS 4 Wireless and Medit i700 Wireless using Wi-Fi transmission) are currently available. These differences primarily concern ergonomics, mobility, and data transmission methods. However, the available literature does not provide clear evidence that the communication method itself (wired versus wireless) significantly affects scanning accuracy. Technical specifications and clinical conditions appear to be more important determinants than connection type.

### 4.4. Clinical and Environmental Factors

Numerous studies have confirmed variability in scan accuracy under clinical conditions, demonstrating reduced performance in the presence of limited working space, subgingival preparation margins, unfavorable optical conditions, and challenging anatomical situations [8,19,22,26,27]. Compared with in vitro procedures, intraoral scanning is characterized by a greater number of local inaccuracies and longer acquisition times. Nevertheless, these deviations generally remain within clinically acceptable limits, supporting the suitability of IOS systems for routine practice [25,26].

Another important methodological aspect concerns scan alignment procedures, as the selection of reference areas during superimposition significantly affects the evaluation of accuracy [28]. Methods based primarily on hard tissues while minimizing soft tissue inclusion are considered the most reliable for long-term analyses.

### 4.5. Clinical Implications and Future Directions

Despite substantial progress in intraoral scanning technology, several important knowledge gaps remain. Most studies evaluated individual variables in isolation, whereas interactions among scanner-related, operator-related, and clinical factors were investigated less frequently. The predominance of laboratory evidence is particularly important: 16 of 19 primary studies were conducted in vitro. Such models permit controlled comparisons but cannot fully reproduce saliva or blood contamination, patient movement, soft-tissue dynamics, restricted intraoral access, or operator–patient interaction. Accordingly, the clinical recommendations derived from this evidence should be regarded as cautious extrapolations until confirmed by well-designed clinical investigations.

The collected evidence clearly indicates that achieving a high-quality digital impression requires comprehensive consideration of all determinants involved in the scanning process. Key factors include appropriate scanner selection, adaptation of the scanning strategy to the specific device and dental arch, maintenance of optimal clinical conditions, and adequate operator training.

Based on the available evidence, and considering the predominance of in vitro studies, several practical considerations may be proposed for clinicians. However, these recommendations should be interpreted as evidence-informed considerations rather than universally validated clinical protocols. Full-arch scans should be performed using a scanner-specific, validated sequence, with particular attention to posterior regions and areas where image stitching may accumulate error. In crowded arches or when scanner maneuverability is limited, careful tip angulation and targeted rescanning of incompletely captured regions may be required. Subgingival finish lines should be adequately exposed, with effective control of moisture and bleeding before scanning. Reflective or translucent restorative materials should be scanned in accordance with device- and material-specific recommendations to minimize optical artifacts. Ambient illumination should remain stable and appropriate for the scanner used, because both light intensity and color temperature may influence geometric accuracy or shade acquisition. These recommendations should not be interpreted as universally validated protocols.

Future research should prioritize the development of standardized study protocols, including harmonized reference measurement methods and accuracy assessment criteria, to facilitate meaningful comparisons across studies. Greater emphasis should also be placed on well-designed clinical investigations evaluating scanner performance under challenging conditions, such as full-arch scanning, subgingival finish lines, extensive implant-supported rehabilitations, and highly reflective restorative materials. Moreover, future studies should investigate the combined effects of operator experience, scanner technology, and environmental factors, rather than considering these variables separately. Finally, the growing integration of artificial intelligence and advanced image-processing algorithms into intraoral scanning systems warrants further investigation to determine their impact on scanning accuracy, efficiency, and clinical decision-making.

Among the analyzed studies, scanner type and scanning strategy were the factors most consistently associated with scan accuracy, whereas the influence of operator experience appeared more variable and system-dependent. A key conclusion is that these factors do not act independently but interact in a complex manner, and the influence of one parameter may be modulated by others.

### 4.6. Additional Clinical Benefits of Intraoral Scanners

Beyond their diagnostic and restorative applications, intraoral scanners provide several additional clinical benefits related to patient comfort and communication. The analyzed studies consistently indicate that IOS systems are better accepted by patients than conventional impression techniques. Patients frequently report improved comfort associated with the absence of impression materials, unpleasant taste, odor, and gag reflex stimulation [29,30].

Digital scanning also appears to enhance communication between clinicians and patients. Three-dimensional visualizations facilitate the explanation of treatment needs, improve understanding of oral health conditions, and may increase patient motivation to participate in treatment. In one study, 94% of patients reported greater motivation to undergo treatment after viewing their intraoral scans [30].

These findings suggest that IOS technology fulfills not only a diagnostic role but also an educational and communication-support function, contributing to improved patient engagement and acceptance of digital workflows in contemporary dental practice.

### 4.7. Comparison with Previous Literature Reviews

The findings of the present review extend previous syntheses of factors influencing intraoral scanning accuracy by incorporating evidence from contemporary intraoral scanning systems. Comparison with previous reviews indicates that although several established determinants remain relevant, their relative contribution may have evolved with advances in IOS hardware and software.

The role of operator experience represents one notable area of change. Cortes et al. identified operator experience as a significant determinant of IOS trueness, consistent with earlier evidence from Abduo and Elseyoufi [5,31]. In contrast, the more recent studies included in the present review produced less consistent findings, with several reporting no significant association between operator experience and scan accuracy [15,16,17,18]. This difference may partly reflect advances in data-processing and registration algorithms, including AI-assisted approaches that facilitate automated segmentation and artifact removal [2]. Nevertheless, operator-related factors remain relevant, particularly in complex clinical situations, while the adverse effects of rescanning and post-processing reported by Cortes et al. remain clinically pertinent [31].

The effect of scan extent appears to be more persistent. The previously described relationship between increasing scan length and cumulative registration errors was also supported by the present review [5,31]. In particular, complex arch morphology, including severe dental crowding, may further compromise image registration [23,24]. Thus, despite technological development, extensive and full-arch scanning remains susceptible to accuracy degradation.

The present review also extends the framework proposed by Alkadi by highlighting the interaction between environmental conditions and the optical properties of scanned surfaces [6]. Recent evidence indicates that ambient illumination may influence scanning performance in a scanner-dependent manner, while restorative material characteristics, including shade, translucency, and reflectivity, may further affect scan acquisition and registration [21,26,27]. At the same time, limitations described in earlier literature, particularly those associated with difficult-to-access preparation margins and intraoral conditions, remain relevant [1,19]. These findings suggest that technological advances may mitigate some operator- and processing-related sources of error, whereas anatomical, optical, and clinical constraints remain important determinants of IOS accuracy.

Overall, the present review complements the work of Cortes et al. by incorporating evidence from the recent era of AI-assisted data processing and contemporary IOS technologies [31]. Rather than replacing previously established determinants, the current evidence suggests changes in their relative contributions, with operator-related effects appearing less consistent, while scan extent, anatomical complexity, material properties, and clinical acquisition conditions remain important determinants of scanning accuracy.

### 4.8. Limitations of the Review

The main limitations of the present review are its narrative design, restriction to two databases and English-language publications, and the absence of a formal risk-of-bias assessment. The included evidence was methodologically heterogeneous with respect to experimental models, reference standards, scanner systems, and definitions of trueness and precision. Of the 19 primary studies, 16 were in vitro, and only three were clinical; therefore, most conclusions describe controlled experimental performance rather than verified clinical effectiveness. The single included review article was used to provide broader contextual interpretation, identify additional technical and environmental considerations, and support the discussion of findings across heterogeneous study designs. However, it was not weighted as equivalent to primary investigations and did not independently determine the conclusions of this review.

Future studies should consider the development of predictive models based on machine learning to estimate scan quality according to combinations of operator-, device-, and environment-related factors. Additional research is also required to evaluate psychosocial and perceptual factors that may influence patient acceptance of digital technologies and the successful implementation of digital protocols in dental practice.

Despite these limitations, the collected evidence enables the formulation of coherent conclusions with practical significance for contemporary digital dentistry.

## 5. Conclusions

The accuracy of intraoral scanning results from the interaction of multiple factors, including scanner type, scanning strategy, operator experience, restorative material, and clinical/environmental conditions.

1. Scanner type appears to be an important determinant of reproduction quality, particularly in complex clinical cases and full-arch scans, but the available evidence does not allow for a universal ranking of scanner performance.
2. The effectiveness of a scanning strategy depends on the specific IOS system and clinical indication, excluding the existence of a universal scanning protocol.
3. The influence of operator experience on accuracy remains inconclusive and appears to be less significant in modern systems, although it still affects scanning time, efficiency, and management of difficult clinical conditions.
4. Clinical conditions such as lighting, arch morphology, limited scanner maneuverability, reflective or translucent materials, and subgingival preparations may affect scan accuracy. Humidity and moisture control should be considered clinically relevant confounders, although direct experimental evidence for humidity as an independent factor was limited in the included studies.

Achieving an optimal digital impression therefore requires individualized adaptation of the scanning procedure to the specific IOS system, clinical conditions, and therapeutic indication. Because the current evidence is predominantly laboratory-based, these recommendations should be applied cautiously and refined as additional clinical data become available.

## Figures and Tables

**Table 1:** Summary of studies evaluating factors affecting intraoral scanner accuracy.

No.	Category	Study (Author, Year)	Methodology	Main Findings
1	Scanning strategy	Ortensi et al., 2024 [10]	In vitro study; 3 scanning strategies (S1–S3); 4 IOS systems.	Scanning strategy significantly affected trueness, precision, and scanning time. S2 showed the highest precision, whereas S3 achieved the highest trueness for selected scanners.
2	Scanning strategy	Grande et al., 2025 [11]	In vitro complete-arch implant study; different implant scan bodies, IOS systems, and scanning strategies.	Implant scan body type and IOS influenced accuracy. Scanning strategy mainly affected efficiency rather than trueness.
3	Scanning strategy	Răuță et al., 2026 [12]	In vitro comparison of Medit i700 and Trios 5 using three scanning strategies.	Both scanner type and strategy affected accuracy. Medit i700 was more precise, whereas Trios 5 scanned faster but with greater variability.
4	Scanning strategy	Liu et al., 2024 [13]	In vitro study; three scanning strategies; maxillary and mandibular scans.	No significant differences for the maxilla. For the mandible, specific strategies reduced deviations depending on the scanner.
5	Scanning strategy	Chang et al., 2023 [14]	In vitro study; three scanning strategies; Trios 3 scanner.	No significant differences in overall accuracy. The TR strategy showed the most favorable distribution of measurement points.
6	Operator experience	Sawangsri et al., 2025 [15]	In vitro study; operators with different experience levels.	Greater operator experience significantly improved trueness and reduced scanning time.
7	Operator experience	Thomas & Jain, 2023 [16]	Prospective clinical study; 20 patients; three operator groups; two IOS systems.	Experience reduced scanning time but had no significant effect on accuracy.
8	Operator experience	Lin et al., 2025 [17]	In vitro study; 30 operators; three IOS systems.	Operator experience did not significantly affect accuracy. Scanner type had a greater influence.
9	Operator experience	Schimmel et al., 2021 [18]	In vitro study; experienced vs. inexperienced operators; edentulous and partially edentulous models.	Experience had only a minor influence on accuracy but improved scanning efficiency.
10	Scanner type & restorative material	Çakmak et al., 2024 [19]	In vitro study; Primescan vs. TRIOS 3; different finish-line locations.	Subgingival margins showed the lowest trueness. Primescan achieved higher trueness, whereas TRIOS 3 provided better cervical margin quality.
11	Scanner type & restorative material	Revilla-León et al., 2023 [20]	In vitro study; four finishing procedures; four IOS systems.	Airborne-particle abrasion provided the highest trueness and precision, whereas immediate dentin sealing showed the lowest accuracy.
12	Scanner type & restorative material	Budati et al., 2026 [21]	In vitro study; three scanning technologies; different crown materials.	Reflective and translucent materials increased scanning errors. Scanner performance depended on material type.
13	Clinical & environmental factors	Yu et al., 2026 [22]	In vitro study comparing intraoral and extraoral scanning conditions.	Restricted intraoral maneuverability reduced scanning performance and increased scanning time.
14	Clinical & environmental factors	Kirschneck et al., 2018 [23]	Clinical study comparing intraoral scanning with plaster model digitization.	Intraoral scanning showed lower conformity in posterior regions and stitching areas.
15	Clinical & environmental factors	Lin et al., 2024 [24]	In vitro study evaluating different degrees of dental crowding.	Severe crowding reduced scanning trueness, whereas interdental spacing improved scan accuracy.
16	Clinical & environmental factors	Shen et al., 2024 [25]	In vitro comparison of intraoral and indirect scanning techniques.	No significant differences were observed in most clinical regions, supporting the reliability of IOS.
17	Clinical & environmental factors	Zhou et al., 2024 [26]	In vitro study; different zirconia shades under various lighting conditions.	Darker zirconia shades required additional illumination to maintain scanning accuracy.
18	Clinical & environmental factors	Guo et al., 2025 [27]	In vitro study evaluating illuminance and color temperature.	Illuminance affected geometric accuracy, whereas color temperature mainly influenced shade matching.
19	Clinical & environmental factors	Fratila et al., 2025 [8]	Literature review of technical, clinical, and environmental factors.	IOS accuracy depends on scanner characteristics, software, tissue morphology, lighting, and clinical conditions.
20	Clinical & environmental factors	Vargas-Quiroga et al., 2025 [28]	Clinical study comparing alignment methods for digital mandibular models.	Alignment methods based mainly on hard tissues provided the highest repeatability and trueness.

## Data Availability

No new data were created or analyzed in this study. Data sharing is not applicable to this article.

## Abbreviations

The following abbreviations are used in this manuscript:

IOS	Digital intraoral scanner
